# Zero electromagnetic coupling of closely spaced identical helical resonators

**DOI:** 10.1038/s41598-026-36975-4

**Published:** 2026-02-07

**Authors:** J. Gudge-Brooke, N. Clow, A. P. Hibbins, A. W. Powell, J. R. Sambles

**Affiliations:** 1 Department of Physics and Astronomy, Centre for Metamaterial Research and Innovation, University of Exeter, Stocker Road, Exeter, EX4 4QL UK; 2 DSTL, Porton Down, Salisbury, Wiltshire, SP4 0JQ UK

**Keywords:** Engineering, Materials science, Optics and photonics, Physics

## Abstract

The interaction between closely spaced elements in an electromagnetic array typically leads to significant inter-element coupling, altering the resonance properties of each element. This coupling influences, often limiting the performance of metamaterials, filters, and phased arrays. In this study using both numerical simulations and experimental validation, we explore the electromagnetic coupling between identical helical microwave resonators and demonstrate how, under specific geometric conditions, near-zero coupling can be achieved even at highly sub-wavelength separations ($<\frac{\lambda }{10}$). Experimental samples are produced using 3D-printed molds subsequently filled with low-melting-point Field’s metal, enabling precise and repeatable resonator construction. The numerical analysis is further extended to infinite periodic chains of identical helices, revealing that similar geometric conditions enable control over propagating mode dispersion, including near-zero group velocity.

## Introduction

We explore how the resonances of two identical helical microwave resonators placed in close proximity can be modified to achieve near-zero electromagnetic coupling by employing specific rotated geometries. This ability to suppress coupling is not only fundamentally interesting, but also practically important for a wide range of electromagnetic and metamaterial applications, including compact antenna arrays, sensors and reconfigurable microwave systems. In such systems, unwanted mutual coupling between adjacent resonators or radiators can degrade performance, alter resonance frequencies, and limit design flexibility. Minimizing inter-element coupling could therefore lead to more design freedom, especially for compact devices. We also show that while the coupling between the two elements is near-zero the resonant frequency is still perturbed from that of an isolated element through higher order interactions.

Strong coupling has been extensively studied and even exploited in wireless power transfer systems and coupled-resonator filters, such as in studies by Kurs et al.^1^ and Tatartschuk et al.^2^. Other works have also aimed to optimize power transfer efficiency between helices^3–5^, however few have focused on the underlying principles that govern their coupling behaviour, or methods to reduce coupling. While some level of coupling is often exploited for useful effects (e.g., band formation in metamaterials), uncontrolled or excessive coupling can disrupt performance. For example, in phased array antennas, mutual coupling between elements can distort radiation patterns, alter input impedance, and degrade array efficiency and beam steering accuracy^6,7^. Similarly in compact resonator arrays, unintentional coupling can shift resonance frequencies, broaden bandwidths unintentionally, or make the electromagnetic response sensitive to small fabrication tolerances^8^.

Despite these challenges, mutual coupling is often treated as an unavoidable side effect, and strategies for systematically minimizing or controlling it are comparatively rare. Addressing this issue is crucial as device miniaturization trends continue; being able to suppress coupling without increasing element spacing would enable more predictable and reliable designs in tightly packed systems. Identifying and engineering conditions such as zero-coupling points thus represents a powerful tool for advancing compact electromagnetic technologies.

Zero coupling refers to the condition where adjacent resonators do not exchange energy, resulting in no collective interaction or phase correlation between them. In this case, each element oscillates independently at its natural frequency, and the overall dispersion relation becomes flat, indicating zero group velocity. Physically, zero coupling is often defined not for elements placed closely together, but for structures that are sufficiently far apart that their near fields do not overlap. Under such separation, mutual inductance or capacitance becomes negligible, effectively eliminating any electromagnetic coupling between neighboring units.

A similar zero-coupling phenomenon has been reported in the context of split-ring resonators when arranged in specific spatial configurations^9^, though this effect has not been widely explored in helical systems. In the case of split-ring resonators, the structures were treated as purely magnetic elements and modeled using equivalent LCR circuits. In contrast, helices exhibit more complex electromagnetic responses, combining electric and magnetic dipole contributions^10^ while also allowing significantly sub-wavelength structures.

Prior studies, such as those by Tak et al.^11^ investigated how the coupling changes with helix spacing. Petrov et al.^12^, demonstrated that careful control of orientation and positioning can lead to suppression of coupling between helices, however this work focused on how electric and magnetic coupling changed with helix arrangement, and did not explore the impact of near-zero coupling configurations on the resonance conditions, or the electric and magnetic fields of the helices. There has also been little study into how this coupling behavior impacts longer chains of independent resonators.

Here, we demonstrate both numerically and experimentally that by rotating a pair of identical helices about a specific axis through their center of symmetry, the two lowest-order coupled modes can be brought into degeneracy, indicating from a simple model, near-zero coupling. The capability to suppress coupling without increasing the element spacing provides an effective means for designing densely packed electromagnetic systems with minimal mutual interference. We extend this concept to periodic chains of helices, enabling control over dispersion characteristics and provide further insight into the role of next-nearest-neighbor interactions when nearest-neighbor coupling is suppressed.

### Coupling

**Fig. 1:**
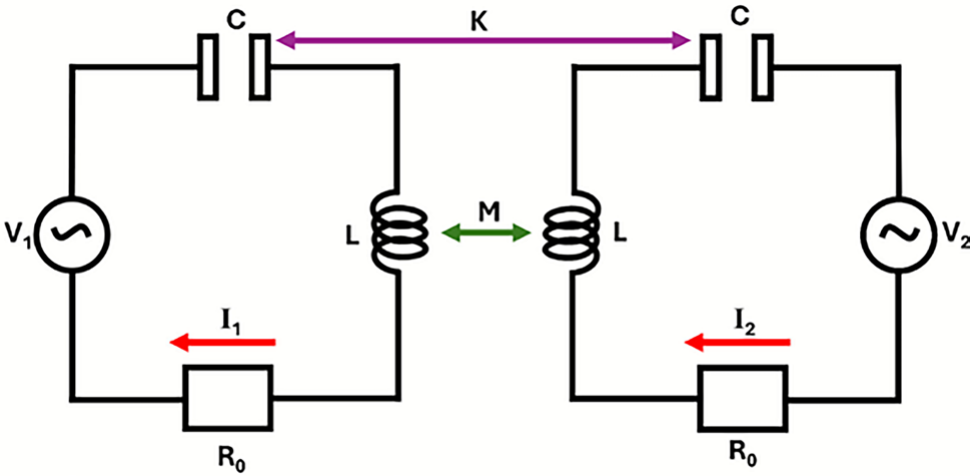
Depiction of equivalent circuit for two coupled helices where: *L* represents the self-inductance of each individual helix, and *C* denotes the self-capacitance. The electrical resistance is given by $R_0$. The driving voltages applied to each helix are $V_1$ and $V_2$, with the corresponding currents $I_1$ and $I_2$. Additionally, *M* and *K* represent the mutual inductance and mutual capacitance, respectively.

One simple way to model coupling between helical resonators is to use LCR circuit theory^13^, for the case of helices that are significantly smaller than the operational wavelength. This approach has been applied to find the coupling coefficients of split-ring resonator by Tatartschuk et al.^2^ and has also been applied to helices by Petrov et al.^12^. These studies use the LCR model of two coupled elements as depicted in Fig. 1.

Using Kirchhoff’s voltage law^14^, the following equations are obtained,

1 $$\begin{aligned} & Z_0 I_1 - i f M I_2 - \frac{1}{if K} I_2 = V_1, \end{aligned}$$

2 $$\begin{aligned} & \quad Z_0 I_2 - if M I_1 - \frac{1}{if K} I_1 = V_2 \text { and} \end{aligned}$$

3 $$\begin{aligned} & \quad Z_0 = R_0 - if L - \frac{1}{if C} = -if L \left( \frac{if_0}{Q} + 1 - \frac{f_0^2}{f^2} \right) . \end{aligned}$$

Here *L* is the self-inductance, *C* is the self-capacitance, and $R_0$ is the electrical resistance of each helix. The driving voltages are $V_1$ and $V_2$, with corresponding currents $I_1$ and $I_2$. Mutual inductance and capacitance are represented by *M* and *K*, respectively. The impedance of each helix is $Z_0$, its quality factor is *Q*, and the single helix resonant frequency is given by $f_0 = \frac{1}{\sqrt{LC}}$ (in reality helices have a family of resonant frequencies but for this approximate *LC* model we are just using the fundamental).

Magnetic coupling between helices occurs when the magnetic field from the current in one induces a voltage in the other through mutual inductance. This interaction can enhance or reduce the overall coupling, depending on the phase of the fields. This magnetic coupling ($\kappa _\textrm{H}$) term may be written as^12^,

4 $$\begin{aligned} \kappa _\textrm{H}= \frac{2M}{L}. \end{aligned}$$

Similarly the electric coupling term ($\kappa _\textrm{E}$) may be written as,

5 $$\begin{aligned} \kappa _\textrm{E}= \frac{2C}{K}. \end{aligned}$$

The total coupling is then,

6 $$\begin{aligned} \kappa = \kappa _\textrm{H}-\kappa _\textrm{E}. \end{aligned}$$

The choice of whether the coupling coefficients are positive or negative is arbitrary, but chosen in this form to conform with LC circuit theory where the voltages and currents have a 180$^\circ$ phase difference between them. This equation shows the possibility to obtain zero coupling for a system even when the electric and magnetic coupling are strong, so long as they are equal. This leads to a counter-intuitive result where resonators may be in very close proximity but are not perturbing each other.

This theory presumes that the coupling strength is small compared to the self-resonant frequency, such that higher-order interactions and radiation effects can be neglected.

When two resonators are coupled, the coupled system will have both in-phase and out-of-phase modes. For two helices of the same handedness, the in-phase resonance is characterized by electric fields, magnetic fields, and currents in each their aligned in the same direction at their respective peaks in phase. In contrast, the out-of-phase resonance has electric fields and magnetic fields, and currents, oriented in opposite directions in each helix.

Equations 1, 2 and 3 give a total coupling using the approximation of small coupling values of

7 $$\begin{aligned} \kappa =2 \frac{f_1 - f_2}{f_0}, \end{aligned}$$

^15^ where $f_1$ and $f_2$ are the coupled resonant frequencies and $f_0$ is the resonant frequency of one of the elements in isolation.

A higher in-phase resonant frequency ($f_{\textrm{ip}}$) indicates positive total coupling, while a higher out-of-phase resonant frequency ($f_{\textrm{op}}$) signifies negative total coupling. To reflect this a modified version of equation 7 is used

8 $$\begin{aligned} \kappa =2 \frac{f_{\textrm{op}} - f_{\textrm{ip}}}{f_0}. \end{aligned}$$

By exploring the field symmetry and through knowing the mode frequencies using equation 8 we can determine the overall the sign and strength of the coupling (though not the individual electric and magnetic components).

### Coupled helical pairs

**Fig. 2:**
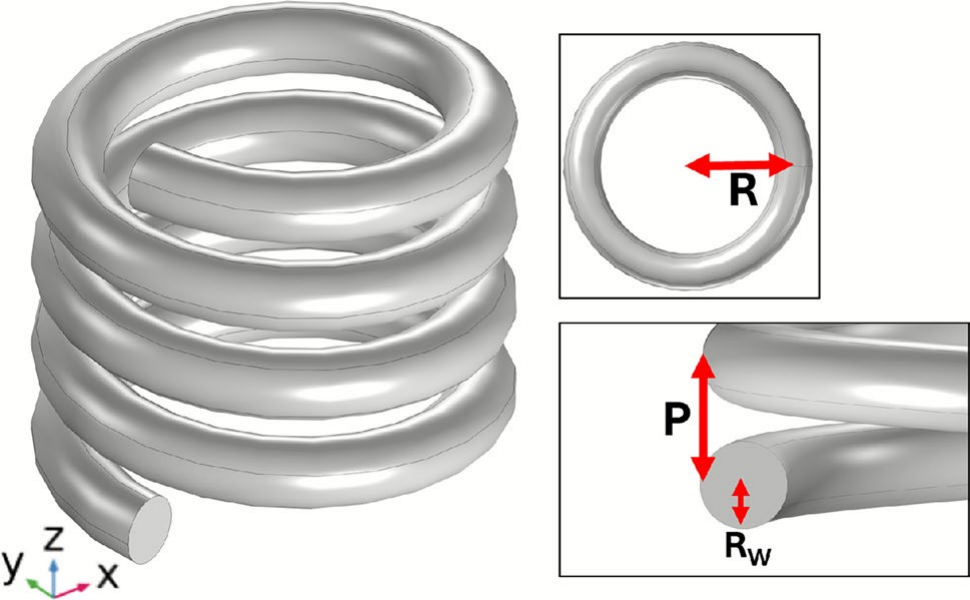
Single handed helix geometry, *R* is the turn-radius from axial center to wire center, $R_\textrm{W}$ is the wire radius, *P* is the pitch.

For this study, a four-turn right-handed helix is used. Throughout the paper, simulations are based on a helix constructed from $R_\textrm{W}$ = 0.7 mm uncoated copper wire with *R* = 4 mm and *P* = 2 mm, as shown in Fig. 2. The pitch is defined as the axial distance corresponding to one full 360-degree turn of the helix.

**Fig. 3:**
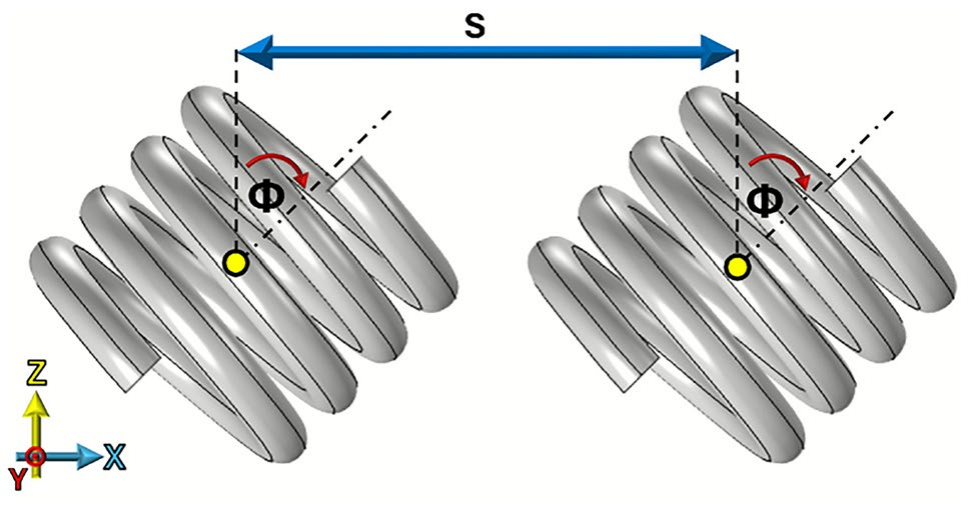
Pair of right handed 4 turn, *P* = 2 mm , *R* = 4 mm, $R_\textrm{W}$ = 0.7 mm copper wire helices with a 14 mm separation (*S*) and a rotation (tilt) of $\varPhi$.

Figure 3 illustrates the geometry used to define both the separation and rotation angles. The separation (*S*) refers to the distance between the central points of the two helices, while the rotational angle is the angle of tilt from the z axis about y. All simulated results are obtained using a finite-element method (FEM) numerical model^16^ using an eigenfrequency solver.

**Fig. 4:**
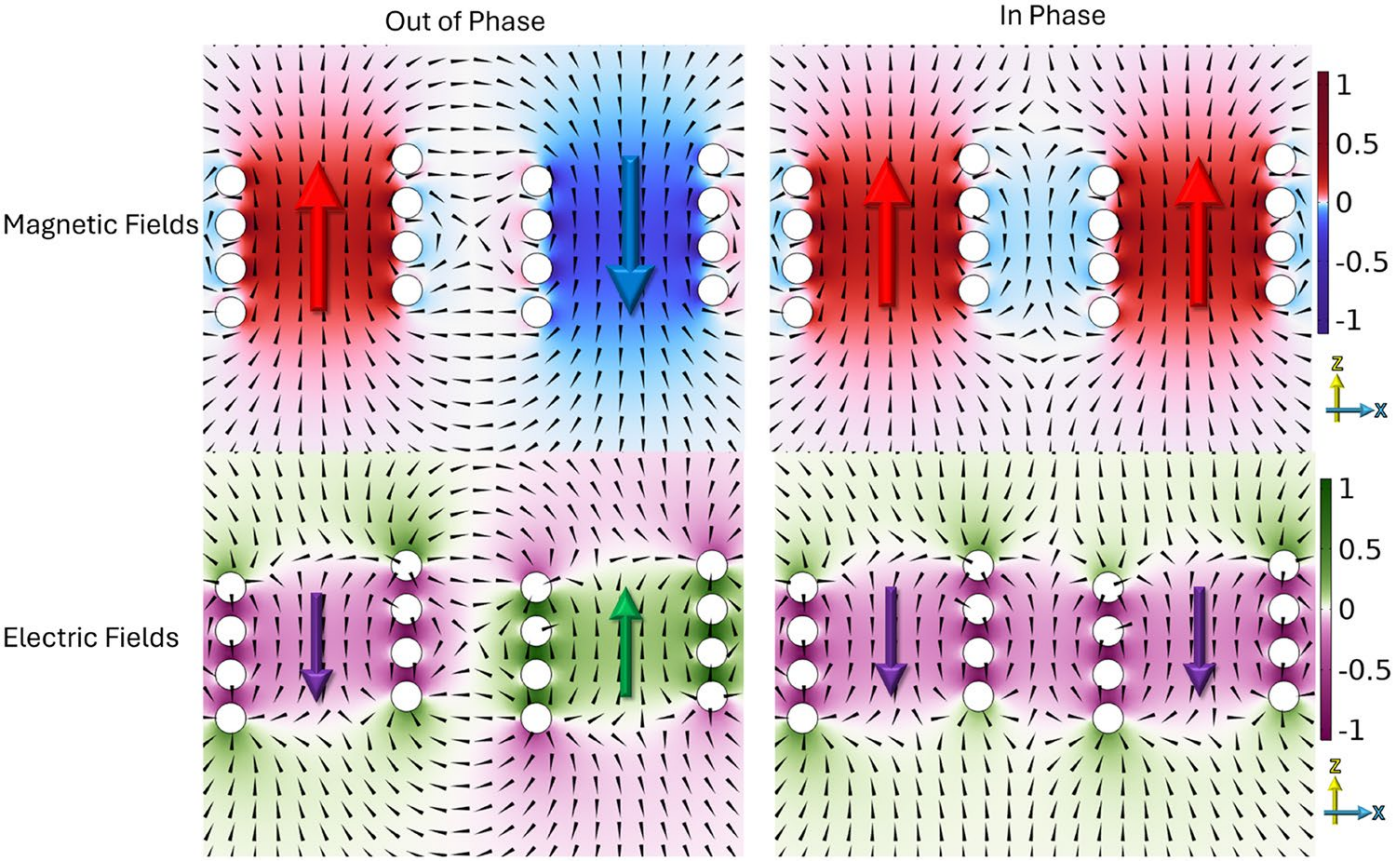
FEM model predictions of the normalized electromagnetic fields in the z-direction in the xz plane at respective phases of peak field of two left-handed 4 turn, *P* = 2 mm, *R* = 4 mm, $R_\textrm{W}$ = 0.7 mm copper helices with *S* =14 mm, $\varPhi$ = 0. $f_{\textrm{ip}}$ at 1.23 GHz and $f_{\textrm{op}}$ at 1.17 GHz.

The electric and magnetic field configurations of the two lowest-order coupled modes, when the helices are parallel aligned along the z-axis and separated along the x-axis, are shown in Fig. 4. In this simulation the $f_{\textrm{ip}}$ = 1.23 GHz and $f_{\textrm{op}}$ = 1.17 GHz. These field plots reveal the spatial distribution and symmetry of each mode, and allow classification based on the direction of the electric and magnetic fields through the center of the helices, as well as the associated current distributions along the wires.

For the in-phase mode, the currents and resulting electric and magnetic fields in both helices look similar in each helix. This field distribution leads to increased antagonistic fields and has a higher frequency due to it having a higher energy. In contrast, the out-of-phase mode features electric fields and magnetic fields that are reversed in each helices. This antisymmetric configuration allows the magnetic fields to circulate up one helix and down the other, resulting in a lower energy state. When the out-of-phase resonance is at lower frequency compared to the in-phase resonance the system has negative coupling.

These distinct field characteristics form the basis for understanding the coupling behavior explored in subsequent sections.

**Fig. 5:**
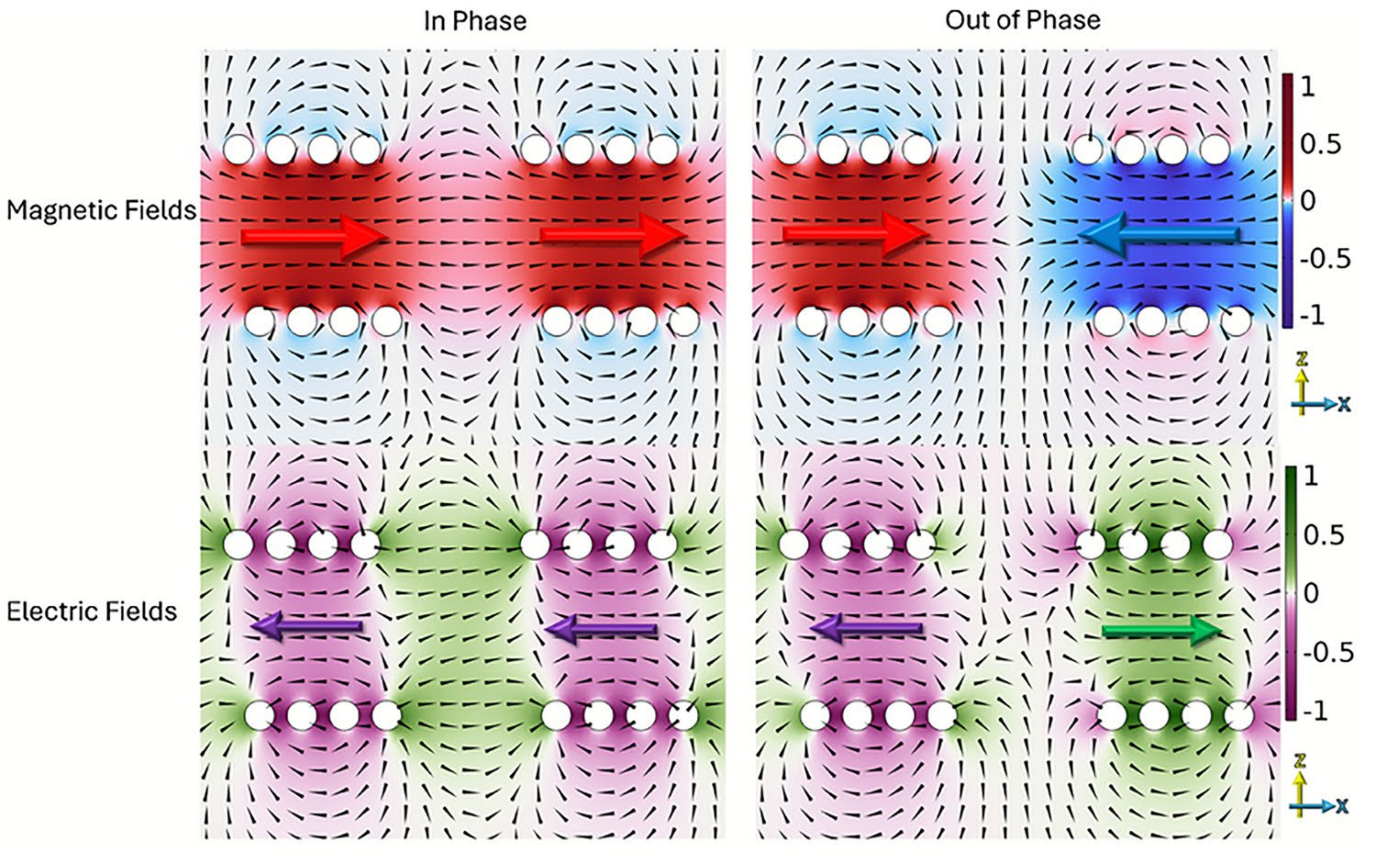
FEM model predictions of the normalized electromagnetic fields in the x-direction in the xz plane at respective phases of peak field of two left-handed 4 turn, *P* = 2 mm, *R* = 4 mm, $R_\textrm{W}$ = 0.7 mm copper helices with *S* = 14 mm, $\varPhi$ = 90. $f_{\textrm{ip}}$ at 1.16 GHz and $f_{\textrm{op}}$ at 1.25 GHz.

The axial aligned configuration is shown in Fig. 5. Now $f_{\textrm{ip}}$ = 1.16 GHz and $f_{\textrm{op}}$ = 1.25 GHz.

**Fig. 6:**
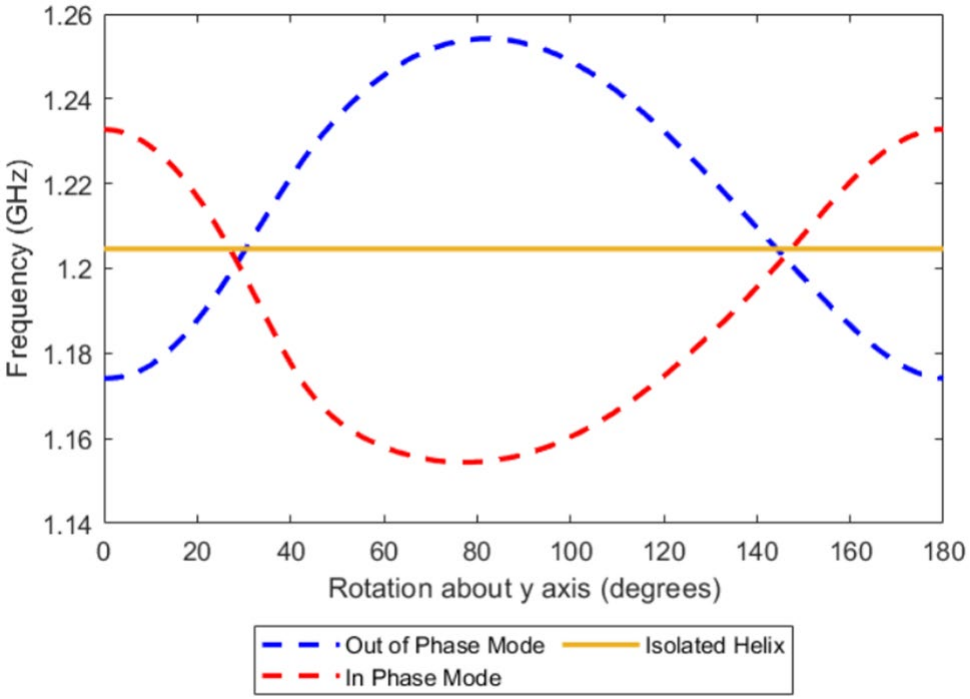
FEM model of the variation of the lowest two order resonant frequencies for two left handed 4 turn, *P* = 2 mm, *R* = 4 mm, $R_\textrm{W}$ = 0.7 mm copper helix and *S* = 14 mm as both are rotated about the y axis.

Thus we see for this spacing when the two helices of the same handedness are aligned along the z-axis at $\varPhi$ = 0 degree rotation, they exhibit negative coupling, which transitions to positive coupling when both helices are rotated by 90 degrees about their centers. To investigate how the two coupled resonant modes evolve with this rotation, a simulation was conducted in which the helices were incrementally rotated, and the resulting mode frequencies were determined as shown in Fig. 6.

Due to the helicity of the structure and lack of rotational symmetry except at $\varPhi$ = 0 and $\varPhi$ = 180, the system does not exhibit perfect symmetry around the 90-degree rotation point. Notably, the simulation reveals two specific angles at which the resonant frequencies of the two modes are equal. This behavior as described by the simple model Eq. 7, indicates that when the resonant frequencies are degenerate, the coupling coefficient $\kappa$ is zero and there is no-coupling between them.

Within the LCR coupling framework discussed earlier, the total coupling between adjacent resonators arises from the combined effects of electric and magnetic interactions. When these two contributions are equal in magnitude and of opposite sign, they cancel each other, resulting in an overall zero-coupling condition as indicated by the crossing point. However this simple LCR model only accounts for the coupling through the lowest order mode.

One important thing to note is that at the frequency at which these two modes converge (1.201 GHz) is below the frequency of a single helix in isolation which is 1.204 GHz. This difference shows that while the model indicates zero-coupling, since the modes have converged in frequency, there is still some interaction between the resonant elements.

In order to explain how the elements interact despite the lowest two modes converging in frequency we look at the coupling for this system at higher frequencies and higher modes to see if the rotational frequency dependence follows the same trend.

**Fig. 7:**
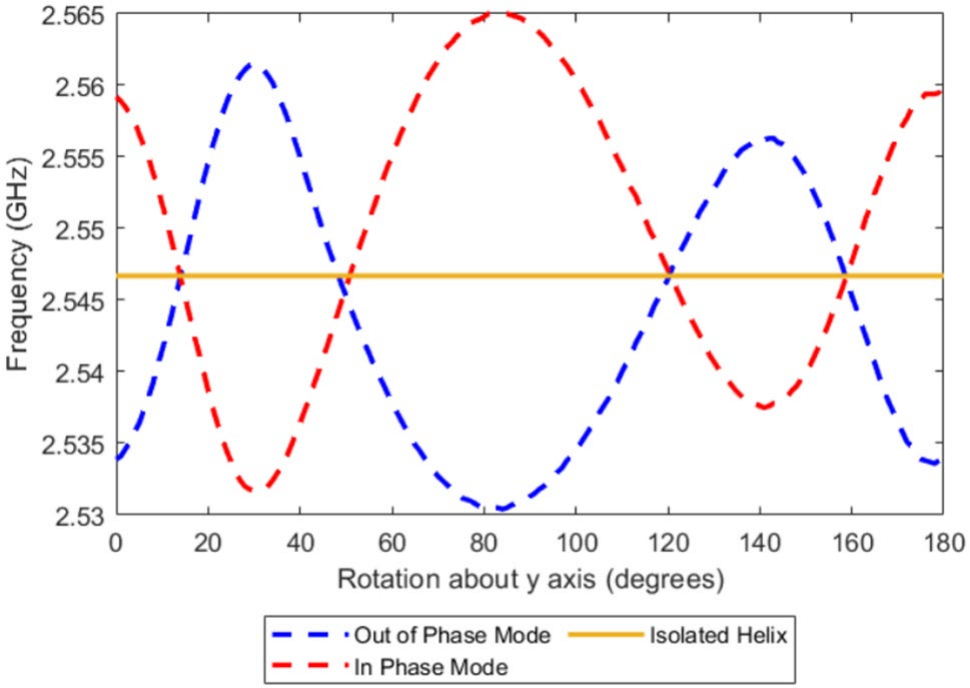
FEM model of the third and fourth order two resonant frequencies for two left handed 4 turn, *P* = 2 mm, *R* = 4 mm, $R_\textrm{W}$ = 0.7 mm copper helix and *S* = 14 mm as both are rotated about the y axis.

Figure 7 shows the rotational dependence of the resonant frequencies for the next-order coupled modes, it also includes the resonant frequency of the second order mode of an isolated helix. In contrast to the lower-order pair of modes which are dipolar, these second-order modes are characterized by quadrupolar field distributions and exhibit a more complex coupling behavior, with four crossing points rather than two.

We note that the degenerate frequencies are offset from the second-order resonance observed in an isolated helix, again indicating that inter-element coupling at converged resonances also plays a role for these higher modes. There are now four rotation angles at which these converge and none occur at the same angle as the lower order convergences, since the coupling behavior of each mode family is governed by different field geometries.

The slight misalignment between the mode crossing of the lowest-order modes and the resonance frequency of an isolated helix can be attributed to the influence of higher-order modes. These higher-order modes do not share the same zero-coupling orientation as the fundamental coupled modes, and their weak but finite interactions slightly perturb the crossing point where perfect cancellation would otherwise occur. This perturbation is minimal and negligible compared with the overall coupling strength away from the crossing angle, although a small residual interaction will still remain. Consequently, it is not possible to achieve truly zero coupling purely through geometric rotation, unless all mode orders exhibit coincident zero-coupling conditions at the same orientation.

The position of the low-coupling point can be tuned by adjusting parameters such as the spacing between resonators. However, increasing the separation uniformly reduces the overall coupling strength at all orientations, making such variations less insightful for this study. Modifying the resonator geometry also shifts the crossing point, as both the individual resonance frequencies and the mutual coupling fields are affected. Nevertheless, preliminary investigations we have undertaken indicate that as long as the lower-order coupled mode corresponds to the out-of-phase resonance at $\varPhi = 0$ and the primary coupling is magnetic, a distinct crossing point persists, signifying the presence of a low-coupling condition.

This highlights that, although the lowest two pairs of modes can exhibit degenerate modes at specific angles, higher order modes are again influencing the interaction, and so the system’s overall response. These interactions shift the resonant frequencies from those expected for isolated elements, and this will be expected for any pair of coupled modes.

## Experimental verification

To explore the coupling between identical helices an alternative fabrication method was employed in place of the traditional approach of winding copper wire in order to enhance consistency across the two resonators. This method involves the creation of a mold that defines the geometry of the resonant structure, which is then filled with metal.

The molds were produced using an Ultimaker S5 3D printer^17^, using PLA (polylactic acid) due to their print-ability and low impact on the target frequencies. This technique offers rapid, repeatable, and highly customisable fabrication compared to wire-wound alternatives.

After printing, the molds were filled with Field’s metal^18^, a low-melting-point alloy with a conductivity of $1.92 \times 10^6 \, \text {S/m}$ at room temperature. Its melting point of 61$^\circ$C and low viscosity make it well suited for injection into fine mold cavities using a hypodermic needle, without damaging the mold material^19^.

**Fig. 8:**
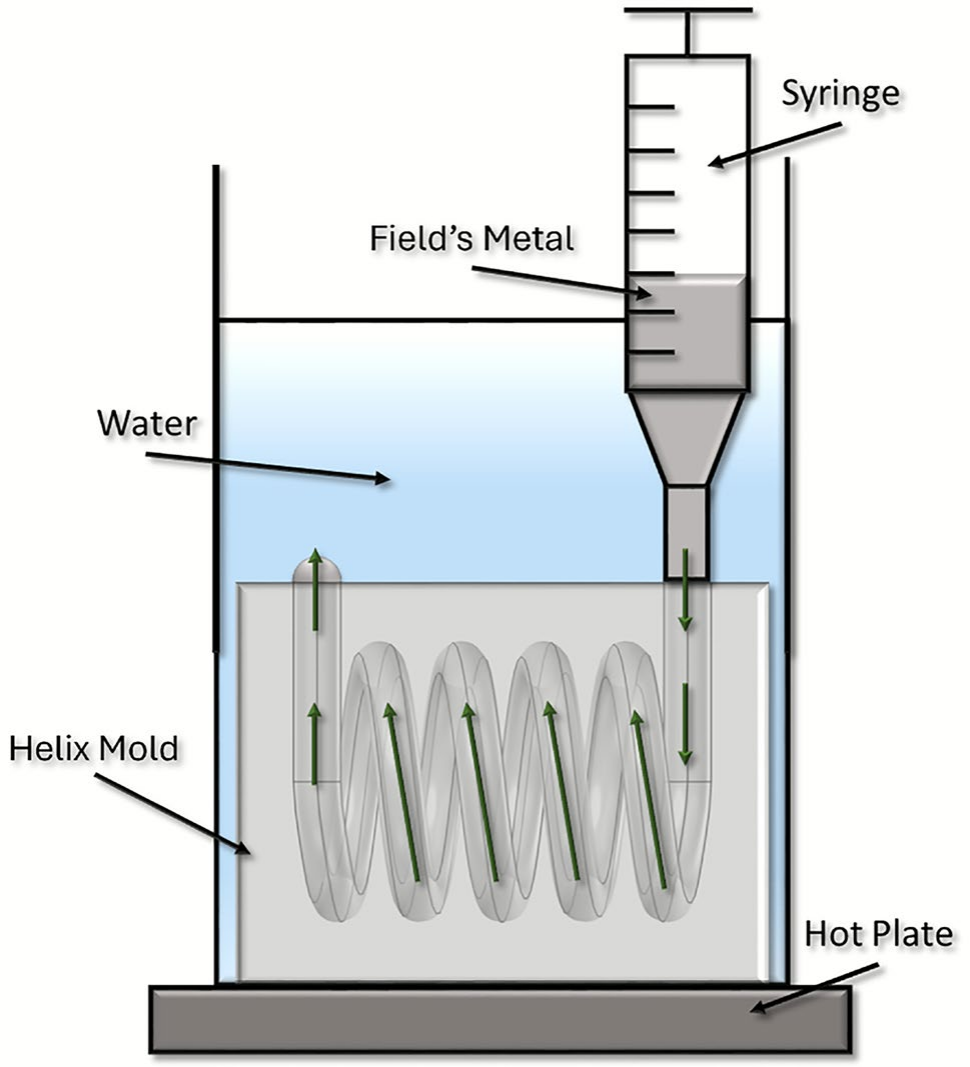
Field’s metal filling technique.

As illustrated in Fig. 8, both the Field’s metal and mold are submerged in a water bath maintained at 61$^\circ$C during filling. The helix molds are designed with an inlet and outlet, enabling complete filling and air displacement. Due to requiring the inlet and outlet to be on the same side the helices fabricated are 4.5 turns rather than an integer number of turns. However, this method imposes certain design constraints, such as prohibiting dead-end cavities or highly branched geometries. To fill the helical cavity within the block, the terminating faces at either end of the helix are extended into arms that facilitate the process.

Once the mold is filled and the metal solidifies, the resulting structure is mechanically robust being encased in dielectric.

**Fig. 9:**
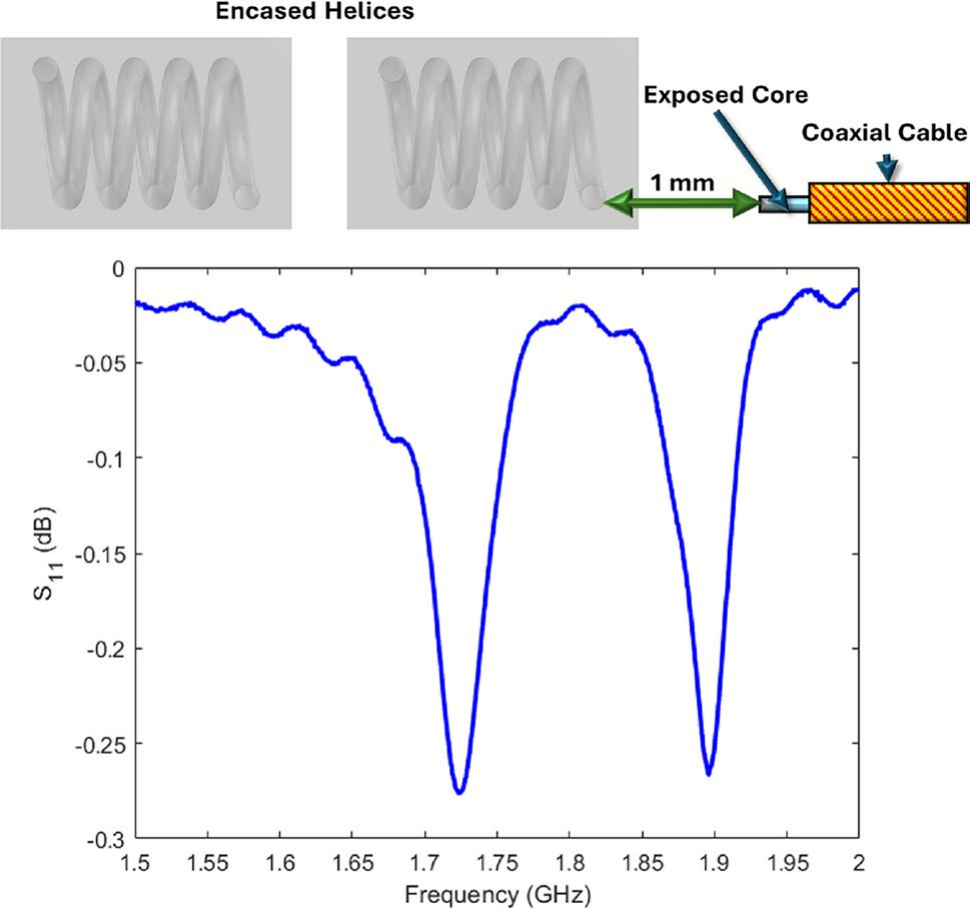
Experimental set up (top) and experimental coupled $S_{11}$ response (bottom) of two left handed helices *P* = 1.4 mm, *R* = 2 mm, $R_\textrm{W}$ = 0.4 mm, *S* = 10.5 mm, $\varPhi$ = 90$^\circ$, 4.8 mm extended arms and 4.5 turns embedded in PLA blocks of width 6.8 mm, depth 6.4 mm, height 9.9 mm excited using a short electric dipole.

The resonances were measured by placing a coaxial probe 1 mm from the end of the helical sample, as shown in the top section of Fig. 9, within an anechoic chamber. The probe was connected to port 1 of a vector network analyzer (VNA), and the reflected signal amplitude ($S_{11}$) was recorded. The $S_{11}$ parameter quantifies the proportion of the input signal amplitude reflected back from the device under test, providing insight into its resonant behavior. To isolate the effect of the sample, the response of the probe in isolation was subtracted from the measured data. The probe itself was constructed from a rigid coaxial copper cable with a 2 mm exposed core. It was intentionally designed to have a resonance well above the frequency range of the samples (around 20 GHz), ensuring minimal interaction or mode overlap with the sample’s resonances. The relative permittivity for the PLA in simulations was 3.9^20^.

Although the use of a dielectric mold reduces the radiative efficiency of the resonant modes, slightly weakening the $S_{11}$ response, the resonances remain clearly identifiable above the noise floor. Since the primary aim is to determine resonance frequencies accurately rather than maximize radiated power, this trade-off is acceptable.

The lower half of Fig. 9 shows the $S_{11}$ response for two encased helices in close proximity with a small excitation probe. This clearly shows two resonant modes allowing for the determination of the frequencies that has then been repeated for several angles of rotation to find the angle of near-zero coupling.

Simulations with dielectric encased helices with the same geometry were conducted for comparison and analysis.

**Fig. 10:**
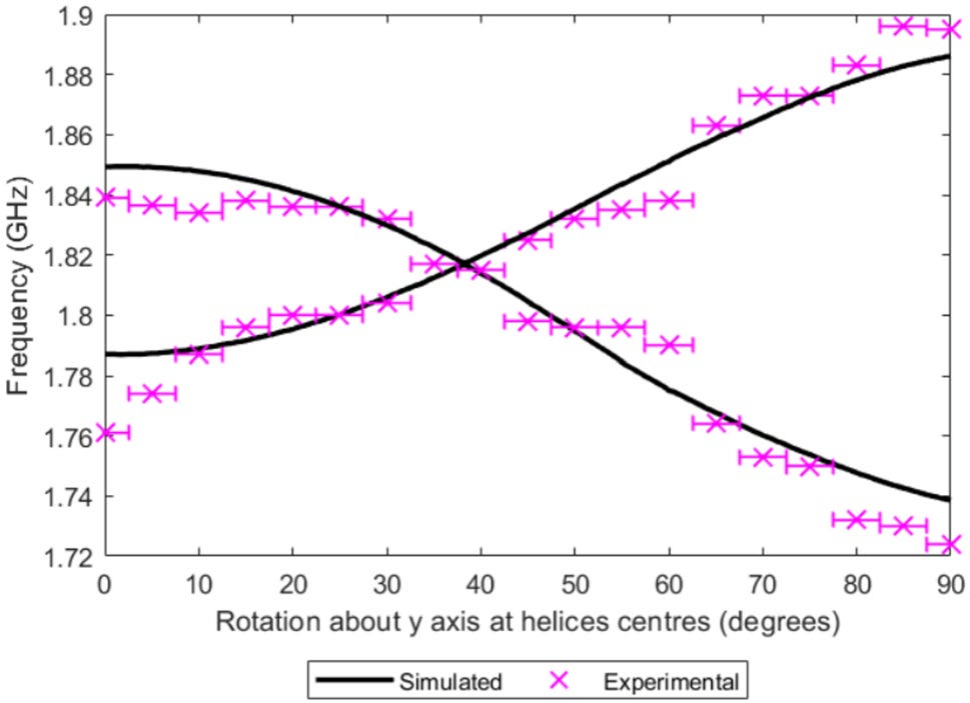
Simulated and experimental measurements of the frequencies of the coupled resonances of two same handed helices *P* = 1.4 mm, *R* = 2 mm, $R_\textrm{W}$ = 0.4 mm, *S* = 10.5 mm, 4.8 mm extended arms and 4.5 turns, embedded in PLA blocks of width 6.8 mm, depth 6.4 mm, height 9.9 mm, excited using a short electric dipole.

As demonstrated in Fig. 10, the measured resonances from the fabricated samples show good overall agreement with the simulated results. These results show, as expected that the modes converge at a specific angle at which they become indistinguishable in frequency as shown in simulated results.

The simulated and experimental results differ primarily due to simplifications in the model, such as assuming a uniform, lossless dielectric with constant permittivity. In practice, 3D printing introduces non-idealities like density variations, microscopic air gaps, and small geometric deviations that influence field distributions. Additional discrepancies arise because the simulated helices are perfectly identical, whereas fabricated structures inevitably contain slight imperfections.

## Infinite periodic array coupling

**Fig. 11:**
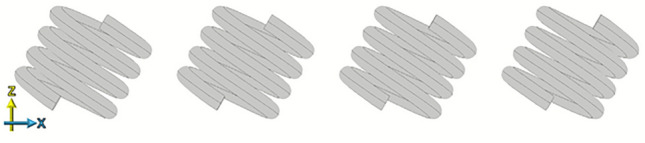
Simulated geometry of a section of the infinite chain of left handed 4 turn, *P* = 2 mm, *R* = 4 mm, $R_\textrm{W}$ = 0.7 mm copper helix with the lattice constant (*A*) = 14 mm.

A natural extension is to expand this study to an infinite periodic array of helical resonators which we model to investigate the mode dispersion of the propagating modes of periodically coupled resonators. A section of which is shown in Fig. 11.

The sign of the coupling between two resonators has a direct influence on the dispersion and group velocity in an infinite chain of helices^21–23^.

For an infinite periodic chain of elements with lattice constant *A*, a simplified dispersion relation becomes^24^:

9 $$\begin{aligned} f(K) = f_0 - \frac{\kappa }{ \pi } \cos (K A), \end{aligned}$$

where *K* is the Bloch wavevector, $f_0$ is the resonant frequency of an isolated resonator and $\kappa$ is the coupling strength defined earlier which can be positive or negative.

This implies the group velocity $v_g$ is:

10 $$\begin{aligned} v_g = \frac{df}{dK} = \frac{\kappa }{ \pi } A\sin (KA). \end{aligned}$$

If $\kappa > 0$ (positive coupling), the dispersion has positive gradient, and $v_g > 0$.

If $\kappa = 0$ (zero coupling) the dispersion will be flat, and $v_g = 0$.

If $\kappa < 0$ (negative coupling), the dispersion gradient is negative, and $v_g < 0$.

The relation above originates from a simplified coupled-resonator model in which each element of the periodic chain interacts primarily with its nearest neighbors. This simplified model does not account for next nearest neighbors as the dispersion is dominated by nearest neighbors. In this, each resonator supports a local oscillation at frequency $f_0$, and magnetic or electric coupling allows energy to transfer between adjacent elements. Applying Bloch’s theorem to this infinite, periodically repeating structure leads to collective oscillation modes (Bloch waves) whose frequencies vary periodically with the Bloch wavevector *K*. In the limiting case of zero coupling ($\kappa = 0$), each resonator behaves independently and the dispersion curve becomes flat at $f_0$, corresponding to zero group velocity.

In such systems this can lead to backward-wave or negative-index phenomena^25,26^.

**Fig. 12:**
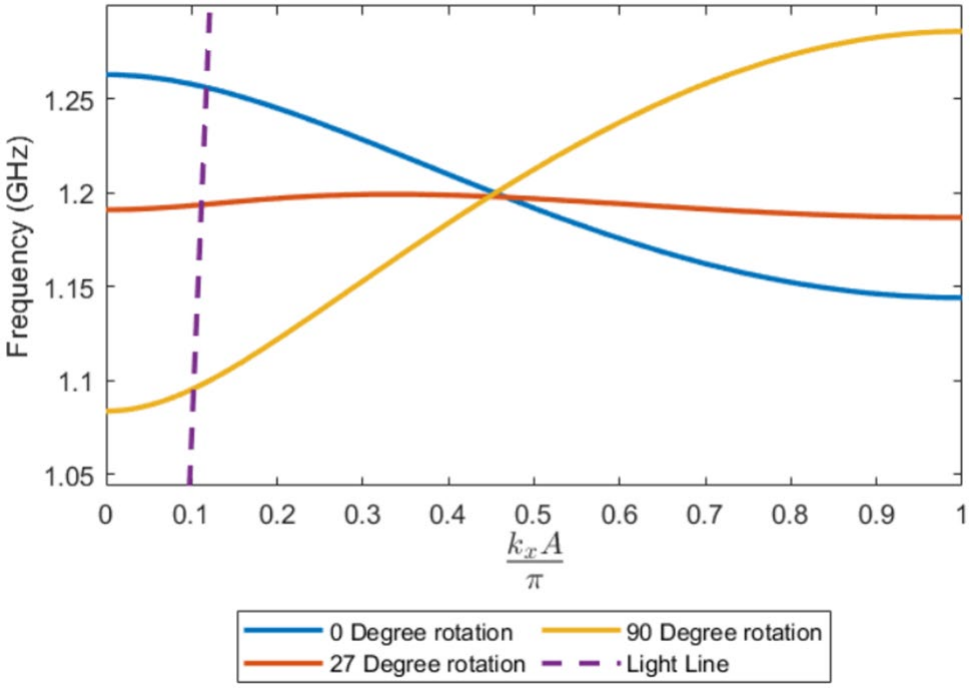
Simulated dispersion of an infinite chain of same handed 4 turn, *P* = 2 mm, *R* = 4 mm, $R_\textrm{W}$ = 0.7 mm copper helix with *A* = 14 mm for three different $\varPhi$ values 0, 27 and 90.

The dispersion relations for this system are presented in Fig. 12 for three different tilts ($\varPhi$). For this model, the unit cell size is defined as $A = 14$ mm. The results were obtained using a single unit cell with periodic boundaries to create a modeled infinitely repeating chain.

The group velocity of the modes is determined, as defined for all waves, from the gradient of the dispersion curve.

When we use an angle that has minimal coupling for two coupled helices (27$^\circ$) there is minimal group velocity. While the frequency is the same value at $\frac{k_xA}{\pi }$ = 0 and $\frac{k_xA}{\pi }$ = 1 there is deviation at the center showing there is still finite group velocity due to each element interacting not only with its immediate neighbor but also with next-nearest neighbors and beyond. These longer-range interactions, though individually weaker, collectively influence the shape of the dispersion relation and prevent perfect flattening of the band unless they too are balanced for every value of $k_x$.

Also note that for the three tilt angles the dispersion curves intersect at $\frac{k_xA}{\pi } \approx 0.47$ with a frequency of 1.2 GHz, which corresponds closely to the fundamental resonant frequency of a single, isolated helix with the same geometry. This value of $\frac{k_xA}{\pi }$ corresponds to where there is half a wavelength per element, matching the field configuration for a pair of single resonant helical elements.

**Fig. 13:**
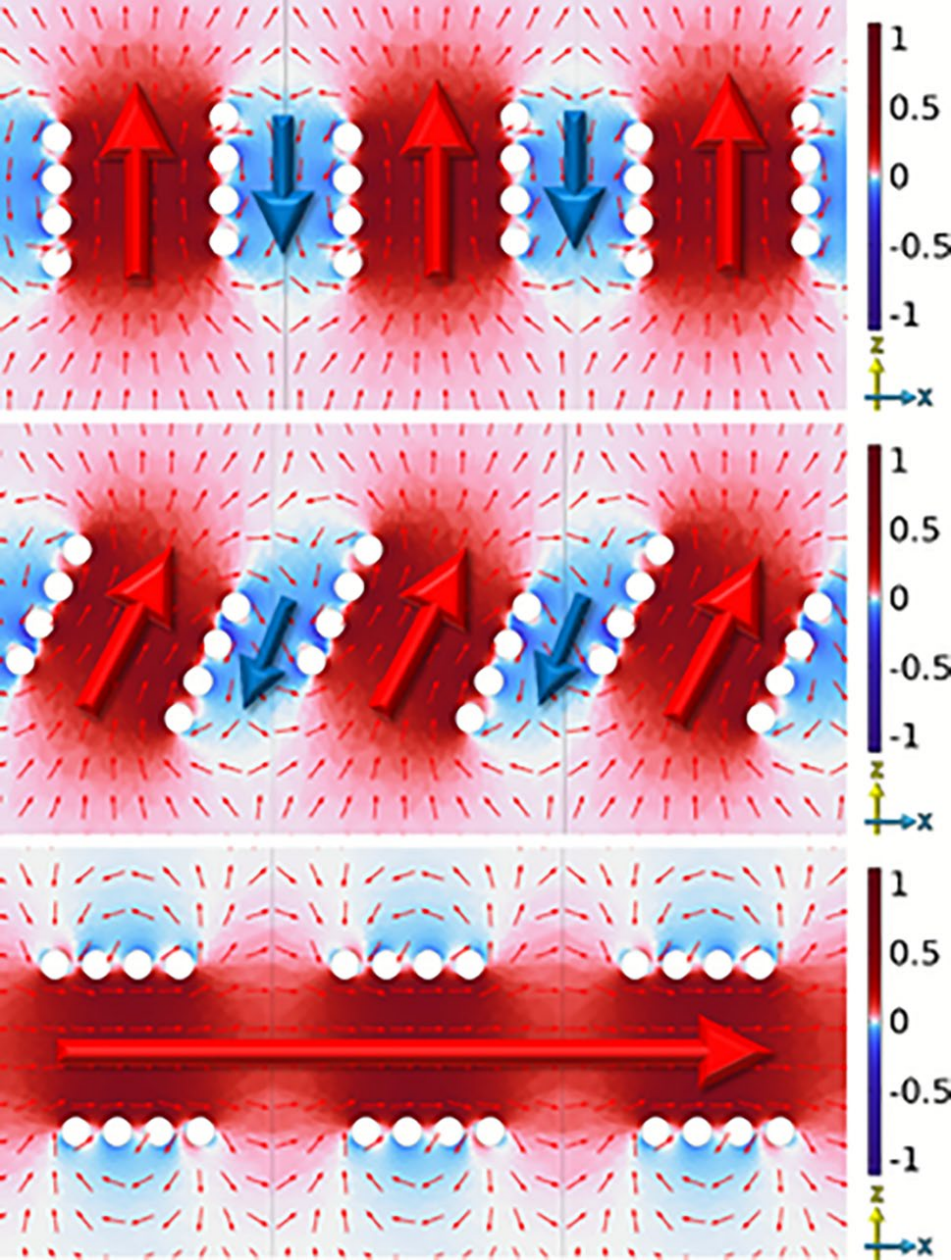
FEM model predictions of the normalized magnetic fields in the direction of the helical axis at respective phases of peak field of infinite chain of left-handed 4 turn *P* = 2 mm, *R* = 4 mm, $R_\textrm{W}$ = 0.7 mm copper helix with *A* = 14 mm, in the xz plane at $\frac{k_xA}{\pi }$ = 0. With $\varPhi$ = 0 (top) at 1.26 GHz, $\varPhi$ = 27 (middle) at 1.19 GHz and $\varPhi$ = 90 (bottom) at 1.08 GHz.

To further explain this behavior in group velocities in Fig. 13 we show the magnetic fields at $\frac{k_xA}{\pi }$ = 0.

The fields at $\tfrac{k_xA}{\pi } = 0$ exhibit distributions similar to the in-phase mode observed in a coupled pair of helices. This explains why the $\varPhi = 0$ configuration has a higher frequency at $\tfrac{k_xA}{\pi } = 0$ compared to $\varPhi = 90$, in the case of a single coupled pair, the $\varPhi = 0$ geometry supports a lower in-phase resonance than the $\varPhi = 90$ configuration.

**Fig. 14:**
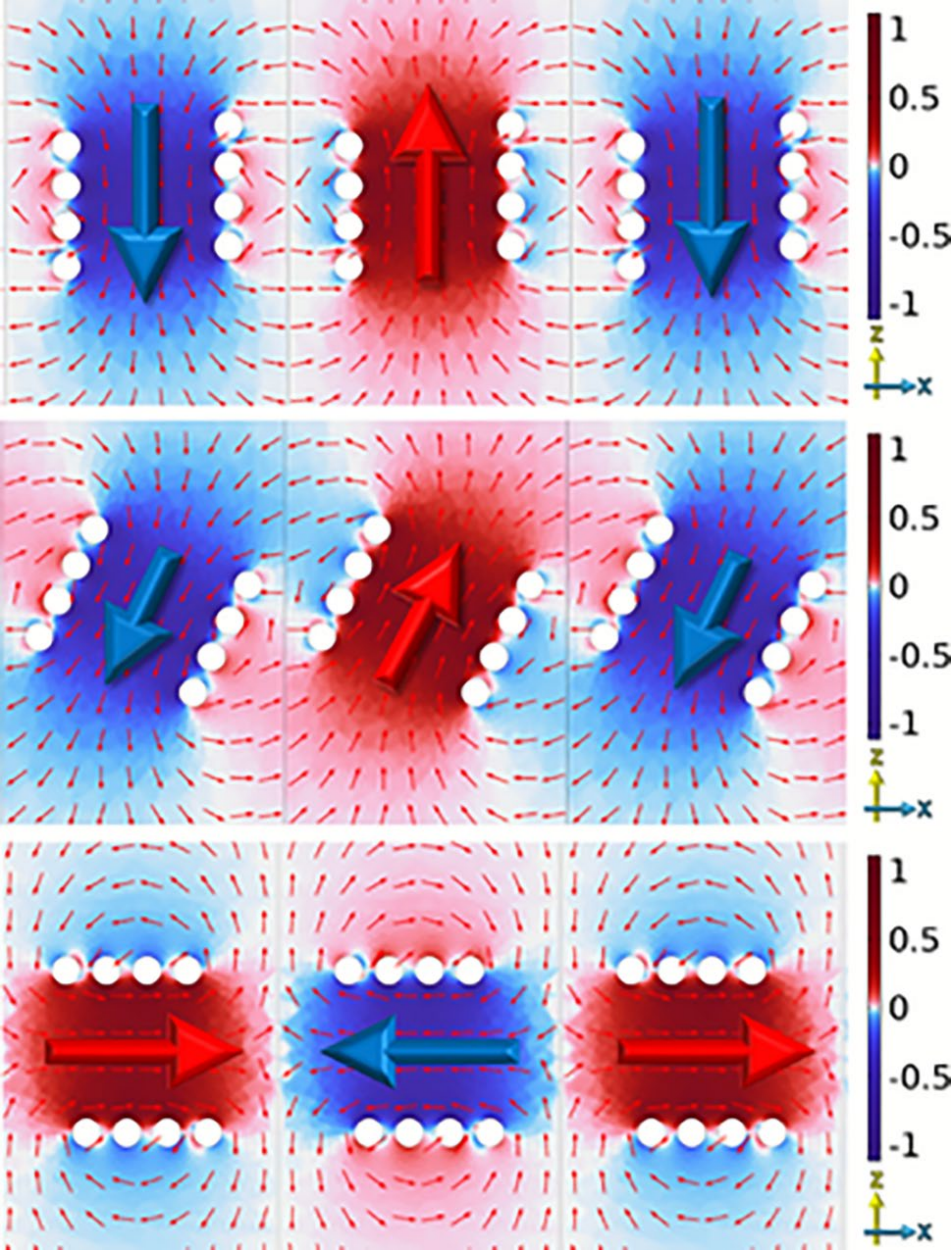
FEM model predictions of the normalized magnetic fields in the direction of the helical axis at respective phases of peak field of infinite chain of left-handed 4 turn, *P* = 2 mm, *R* = 4 mm, $R_\textrm{W}$ = 0.7 mm copper helix with *A* = 14 mm in the xz plane at $\frac{k_xA}{\pi }$ = 1. With $\varPhi$ = 0 (top) at 1.14 GHz, $\varPhi$ = 27 (middle) at 1.19 GHz and $\varPhi$ = 90 (bottom) at 1.29 GHz.

For the fields at $\tfrac{k_xA}{\pi } = 1$ shown in Fig. 14, the situation is reversed, the field distribution corresponds instead to the out-of-phase mode of a coupled pair. Since for a single pair the $\varPhi = 0$ geometry has a lower out-of-phase resonance than $\varPhi = 90$, the frequency ordering reverses at the Brillouin zone boundary.

This transition from in-phase to out-of-phase field distribution between $\tfrac{k_xA}{\pi } = 0$ and $\tfrac{k_xA}{\pi } = 1$ explains why the rotation of the helical resonators changes the ordering of modes.

By changing the tilt of the helices, the frequency ordering of these modes can be reversed, which in turn reverses the slope of the dispersion relation. However we note that since this is a passive system this does not lead to negative group velocity since there is an equivalent negative k region of the dispersion curve with a corresponding positive group velocity.

In contrast, for the weak-coupling configuration at $\varPhi = 27^\circ$, a pair of resonators exhibits nearly degenerate in-phase and out-of-phase resonances. As a result, in the infinite chain there is almost no frequency difference between the mode at $\tfrac{k_xA}{\pi } = 0$ and $\tfrac{k_xA}{\pi } = 1$, leading to a very low group velocity.

The structure presented in this work is primarily intended as a proof of concept to demonstrate the feasibility of guided slow-wave propagation in periodically coupled resonant systems. Its underlying principles are potentially applicable to future meta surface implementations if more precise fabrication and integration techniques are used.

While the results presented here primarily focus on the fundamental dipolar interaction that governs the observed slow-wave behavior, the structure also supports higher-order resonant modes at elevated frequencies. These modes exhibit more complex current and field distributions within each resonator, resulting in less uniform coupling and a reduced ability to sustain low group velocities across most values of $k_x$. For this reason, the present analysis concentrates on the lowest-order (dipolar) resonances, which provides the dominant contribution to slow-wave propagation. It is noted that more intricate geometries or tailored coupling schemes could, in principle, enable the excitation of higher-order modes with controllable dispersion characteristics; however, these effects are comparatively weak and less influential on the overall electromagnetic response than the primary dipolar mode.

The ability to control the sign and magnitude of coupling through geometric alignment allows fine-tuning of energy flow in engineered helical arrays, with direct implications for waveguiding, filtering, and slow-wave applications.

## Conclusion

This study presents an investigation into the electromagnetic coupling between pairs of identical helical resonators at microwave frequencies, focusing on conditions under which near-zero coupling can be achieved. Through both numerical simulation and experimental validation using a novel 3D-printed mold and Field’s metal casting technique, it is demonstrated that rotating two closely spaced helices about their individual center axis enables control over their coupling strength. Notably, specific rotation angles were identified where the two lowest-order resonances become degenerate, indicating a near zero-coupling condition even at highly sub-wavelength separations ($<\frac{\lambda }{10}$).

Beyond the lowest-order dipole modes, higher-order quadrupolar modes were also explored, revealing more complex coupling behavior with multiple zero-coupling points. Extending these findings to an infinite periodic chain of helices, the work further shows that such decoupling conditions lead to low group or even negative propagation, and highlights the interplay between geometry, coupling, and mode symmetry.

These results provide new insight into the design and manipulation of coupled helical systems, with promising implications for applications in metamaterials, compact resonant systems, and near-field communication technologies. The ability to suppress coupling without increasing separation offers a powerful tool for developing densely packed electromagnetic devices with minimal interference.

This paper concerns a one-dimensional chain of resonators; however, the concept could be extended to two- and three-dimensional arrays. Such an extension would require modifications to the resonator geometry to achieve low coupling in multiple directions, as the coupling strength is inherently direction-dependent.

A key application of engineering a zero-coupling point in coupled helical systems lies in the development of reconfigurable or densely packed resonator arrays, such as those used in metamaterials, filters, and phased array antennas, where minimizing mutual coupling is crucial for reducing the overall size of these structures.

## Data Availability

The data that support the findings of this study are available from the corresponding author upon reasonable request.
